# Relational Diversity Promotes Cooperation in Prisoner’s Dilemma Games

**DOI:** 10.1371/journal.pone.0114464

**Published:** 2014-12-04

**Authors:** Bo Xu, Jianwei Wang, Ruipu Deng, Miao Li

**Affiliations:** 1 School of Business Administration, Northeastern University, Shenyang, China; 2 Three Gorges Finance Company Limited, Beijing, China; 3 Department of Engineering Physics, Tsinghua University, Beijing, China; Lanzhou University, China

## Abstract

Relational diversity can be characterized by heterogeneous distributions of tie strengths in social networks and this diversity is present not only among humans, but throughout the animal world. We account for this observation by analyzing two network datasets from Facebook. We measure the strength of a tie by calculating the extent of overlap of friends between the two individuals. Based on the previous findings in human experiments, we argue that it is very unlikely that players will allocate their investments equally to their neighbors. There is a tendency that players prefer to donate more to their intimate friends. We find that if players preferentially allocate their investments to their good friends, cooperation will be promoted in PDG. We proved that the facilitation of the cooperative strategy relies mostly on the cooperative allies between best friends, resulting in the formation of cooperative clusters which are able to prevail against the defectors even when there is a large cost to cooperate. Moreover, we discover that the effect of relational diversity cannot be analyzed by adopting classical complex networks models, because neither of the artificial networks is able to produce networks with diverse distributions of tie strengths. It is of vital importance to introduce real social networks to study the influence of diverse relations especially when it comes to humans. This research proposes a brand new perspective to understand the influence of social relations on the emergence of cooperation in evolutionary prisoner’s dilemma games.

## Introduction

Social relations or social ties are defined as information-carrying connections between people in social networks [1]. Social ties may simply reflect binary relations (the presence or absence of a relation) or they may reflect the strength of a relation between two individuals (e.g. the emotional intensity, the intimacy etc.). Most previous investigations on evolutionary games in social networks adopt the binary definition of the social relation [2]–[4], i.e. A and B are either friends or strangers. Under such definitions, the relationships between friends are universally identical and players will treat all their friends equally. However, social relations or social ties exhibit the feature of diversity in reality. As Granovetter argued, social ties come in three varieties according to tie strengths: strong, weak, or absent [5]. Strong ties exist between close friends or family members, while absent ties denote those relationships without substantial significance, such as “nodding” relationships between people living on the same street. Since most human individuals in social networks socialize with their friends through their social ties, the diverse nature of such social relations will inevitably influence the behavioral traits of interactions between players. Therefore, the strength of social ties should have a significant impact on the evolution of cooperation especially in human social networks. This research tries to unveil the important role of tie strengths in evolutionary prisoner’s dilemma games.

In the classical evolutionary prisoner’s dilemma game (PDG) model [6], each player has two feasible actions: cooperation (C) or defection (D). Both players get R (reward) for mutual cooperation and P (punishment) for mutual defection. A defector exploiting a cooperator gets T (the temptation to defect) and the exploited cooperator gets S (the sucker’s payoff). R, P, T, S satisfies following conditions: T>R>P>S and 2R>T+S. To better illustrate the roles of diverse relations in PDG, we consider an important special case called the “donation game” (DG) [7]–[10] in this paper. In a DG, each player can cooperate by providing a benefit b to the other player at his or her cost c, with 0<c<b. Then, T = b, R = b–c, P = 0, and S = −c. The payoff matrix is (see table 1).

**Table 1:** 

	C	D
C	b–c, b–c	−c, b
D	b, −c	0,0

In the network context, a cooperator’s ability to invest in others is proportional to his or her connectivity 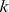. A player is able to donate as much as 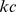 to his or her counterparts in each round. Previous studies assumed that the cooperator will allocate his 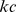 investment equally to his 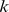 friends without any preferences and each friend will get a benefit b from the investment c. Under such settings, it is suggested that both lattice network and scale free network contribute to the preservation of cooperation in PDG [11]–[14]. Moreover, Santos et. al. found that degree diversity promotes the emergence of cooperation in public good games (PGG) [15]. Since the degree of a node usually represents its relative importance and influence in the social network, several studies also explored the heterogeneous influence of the nodes’ degree on the evolution of cooperation in PDG and PGG [16]–[18]. However, as far as we know, the degree-related influence is not observed in reality. No empirical evidence can support the above degree-related arguments. Instead, researchers have discovered the influence of social relations from empirical experiments, which can be regarded as a solid evidence of the existence of heterogeneous relational influence. The empirical test conducted by Harrison et. al. suggested that the strength of a social tie can predict the cooperative investment in a human social network [19]. The cost endured was positively correlated with the strength of the social tie between donor and recipient. In other words, a cooperator is more willingly to invest in his or her close friends rather than those with only nodding relations [20]. For example, parents would sacrifice everything to their children but little to their ordinary neighbors. Investments from cooperators are distributed heterogeneously among their friends and this heterogeneity is related to their diverse relationships, i.e. tie strengths. Inspired by the above empirical observations, we introduce a relation-based investment strategy into the evolutionary PDG model to investigate how such heterogeneous allocation mechanism affects the final outcome of the game.

**Figure 1:**
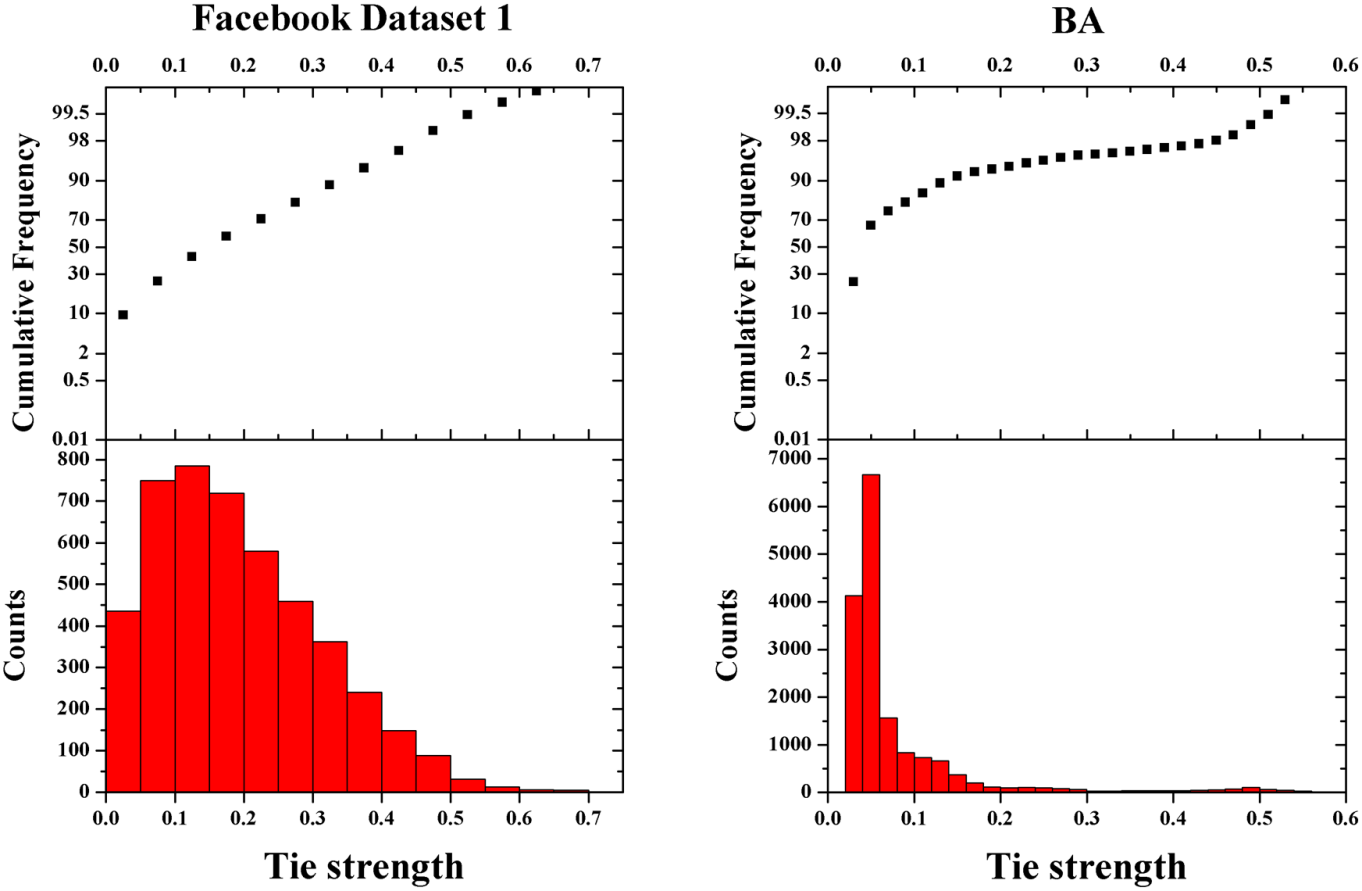
A comparison of the tie strength distributions between a BA network and the network we collected from Facebook.com. The two networks have exactly the same number of nodes and edges. The strength of each tie is calculated by using equation 1. It is obvious that most of the ties in the BA network have approximately zero tie strength, while the Facebook network exhibits a diverse tie strength distribution.

It is worth noting here that we cannot just randomly assign a strength value to a tie in the social network, because numerous previous studies demonstrated that the strength of a tie is associated with its structural position in the social network. Scott et.al. suggested that social embeddedness is a stable structural measure of the strength of a tie in social networks, which is defined as the extent of overlap of social relations between the two individuals [21]. Onnela et. al. [22] studied a mobile phone dataset and defined the aggregated duration of call time between user A and B as the real tie strength between them. Their research also suggested that the stronger the tie between the two users, the more their friends overlap. In other words, the extent of overlapping friends can be a reasonable approximation of the tie strength between two individuals in social networks [23]. The tie strength 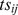 between 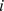 and 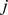 can be calculated as follows:
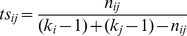
(1)where 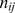 is the number of common neighbors of 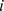 and 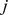, 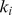 and 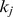 represent the degree of 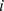 and 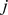 respectively. It is worth mentioning that we will give a minimum tie strength value to a pair of friends if they have no common neighbors (not zero, because they do have a friendship). Another important problem of analyzing the effect of diverse relations is that we cannot adopt classical complex network models (e.g. ER, BA, etc.) [24]–[28] as the structure of the population, because none of these models produces diverse tie strength distributions as we expected in real social networks. To tackle this problem, we use two online social network datasets collected from Facebook.com to perform the analysis. It is illustrated in figure 1 that the real social network we adopted exhibits a more heterogeneous tie strength distribution comparing to other artificial networks such as the BA network.

This paper is organized as follows: In section 2, we give a comprehensive description of the datasets. In section 3, we introduce the model of PDG with a heterogeneous investment strategy depending on tie strengths. Simulation results and discussions are given in section 4.

## Dataset Description

Facebook.com is currently the most popular SNS platform. Users interact by updating personal status, sending private messages, playing desktop games, sharing photos and videos etc. It was initially designed to facilitate communications of college students, and soon became prevalent all over the world. Therefore, the Facebook network can well represent the structure of population in reality.

We plan to perform the analyses on two Facebook datasets with different scales to show that the size of the network has no influence on the conclusion. All datasets are available at http://people.maths.ox.ac.uk/porterm/data/facebook5.zip. A comparison of primary parameters of the two networks is made in table 2. The establishment of friendship in Facebook requires mutual authentication, thus we can infer an undirected graph of its network structure.

**Table 2:** 

	Dataset 1	Dataset 2
Connected vertices	769	6596
Connected edges	11656	293320
Average Connectivity	16.15	44.469
Diameter	6	9
Density	0.0564	0.0135
Average path length	2.3378	2.6761
Clustering coefficient	0.2912	0.1639

**Figure 2:**
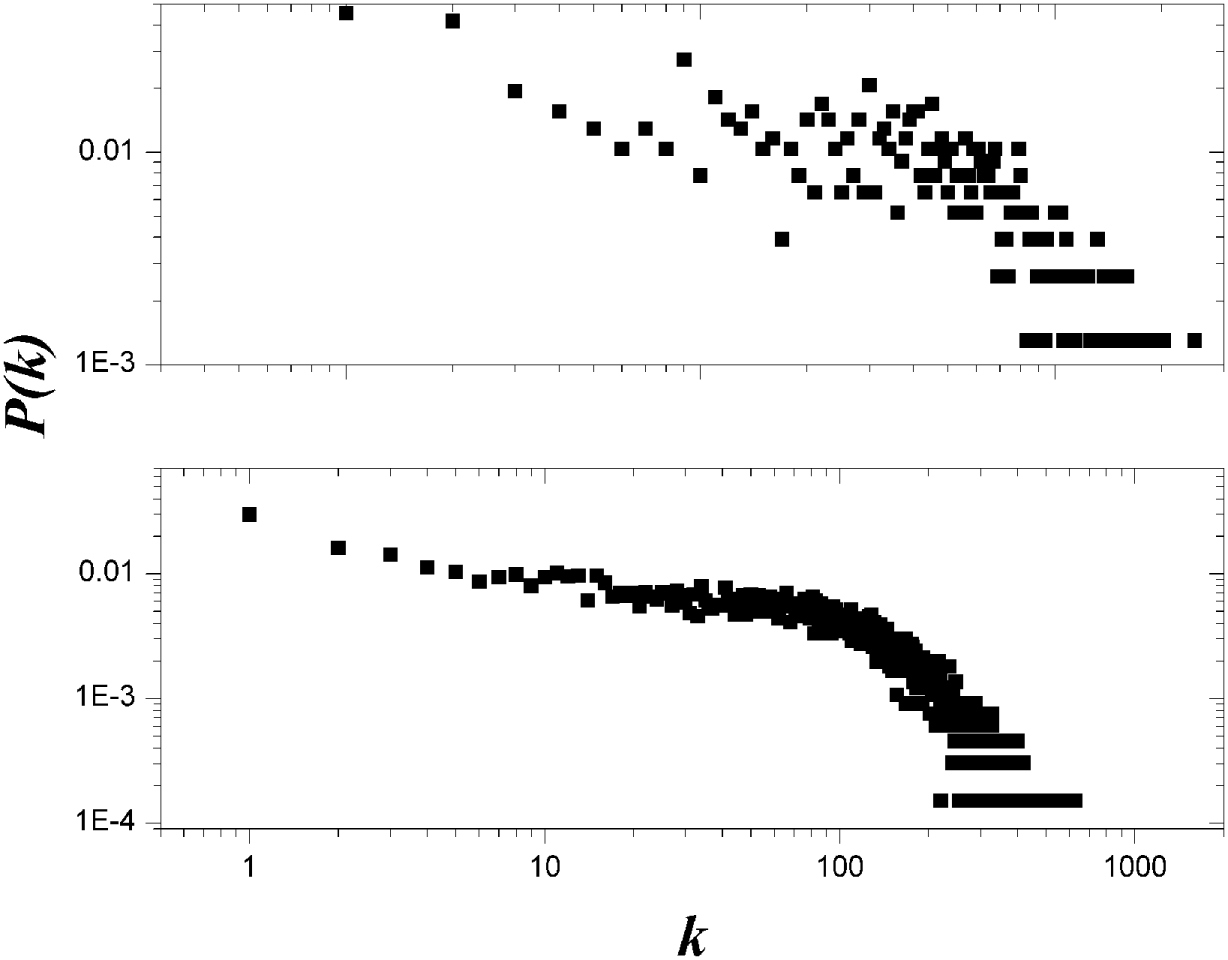
An illustration of degree distributions of the two social networks.

Complex network theory [29]–[31] claimed that most networks in reality (WWW, Internet, DNA, etc.) follow the power law degree distribution, where the probability 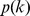 that a randomly selected node having 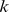 edges obeys the form 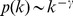. However, figure 2 shows that the degree distributions of the two network datasets do not follow such degree distribution, although they are heterogeneous networks.

## The Model

We assume that the ability of a player in the network is proportional to his or her degree. A cooperator 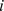 having 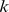 friends has a total amount of 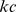 investments in each round and will invest 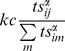 to his friend 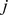. Therefore, the recipient 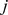 will get 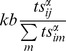 from 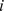
’s investment. Here, 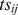 denotes the strength of the tie between the player 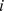 and 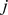, and 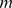 runs over all 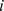
’s friends. 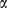 is a tunable parameter controlling the preference of the cooperator. If 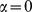, the model becomes a classical PDG. The cooperator will equally distribute his investments and each of his friends gets a benefit 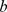. If 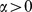, the cooperator will preferentially invest in his good friends. When 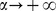, the cooperator will give all his investments to his best friend. The payoff of player 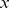 can be expressed as:
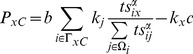
(2)

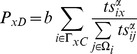
(3)where 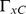 denotes the set of 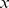
’s friends adopting 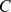 and 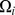 represents the set of 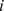
’s friends.

In each round, player 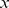 is allowed to adopt the strategy of a randomly selected friend 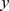 with a probability 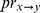 proportional to their payoff difference.
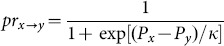
(4)


Where 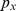 and 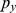 stand for the payoff of player x and y in each round; 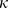 is a parameter characterizing bounded rationality during evolution, 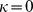 represents complete rationality, 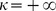 represents complete randomness. We set 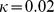 in this study.

## Simulation Results and Discussions

Initially, equal percentage of cooperators and defectors is randomly distributed among the whole population. Strategies of all players are updated simultaneously after each round. The equilibrium fraction of cooperators is obtained by averaging over 1000 generations after a transient time of 10000 generations.

**Figure 3:**
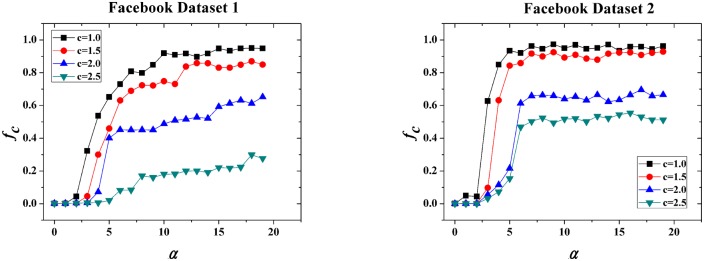
The fraction of cooperators in equilibrium (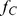) vs 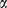 for different values of 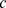. We set 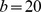 as a constant in all simulations. The two figures show that the increase of 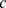 prohibits cooperation since greater 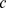 decreases the cooperators’ payoff. Moreover, there is a positive relationship between the preference 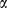 and 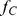 indicating that preferentially allocating investments to good friends promotes cooperation.

The simulation results (see figure 3) show that there is a positive relationship between 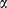 and 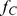, indicating that if cooperators preferentially allocate investments to their good friends, cooperation will be promoted. It is worth noting here that cooperation is prohibited if we adopt traditional PDG models (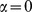) in the above social networks. This result is in agreement with Ohtsuki’s fixation probability theory arguing that high connectivity will suppress cooperation in social networks [7], [32]. Unlike scale free networks, most real social networks are highly dense and clustered. Despite that their degree distributions are indeed heterogeneous, cooperation is still unlikely to emerge in real social networks under traditional settings for the reason that the increasing connectivity will increase the players’ opportunities to play with defectors. Therefore, cooperators are more likely to be invaded by defectors and cooperative clusters will be destructed. Luckily, as we argued above, players are unlikely to treat all their friends equally. The strong social ties enable players to sacrifice more to their best friends, and thus lead to heterogeneous allocation of investments among the entire population.

**Figure 4:**
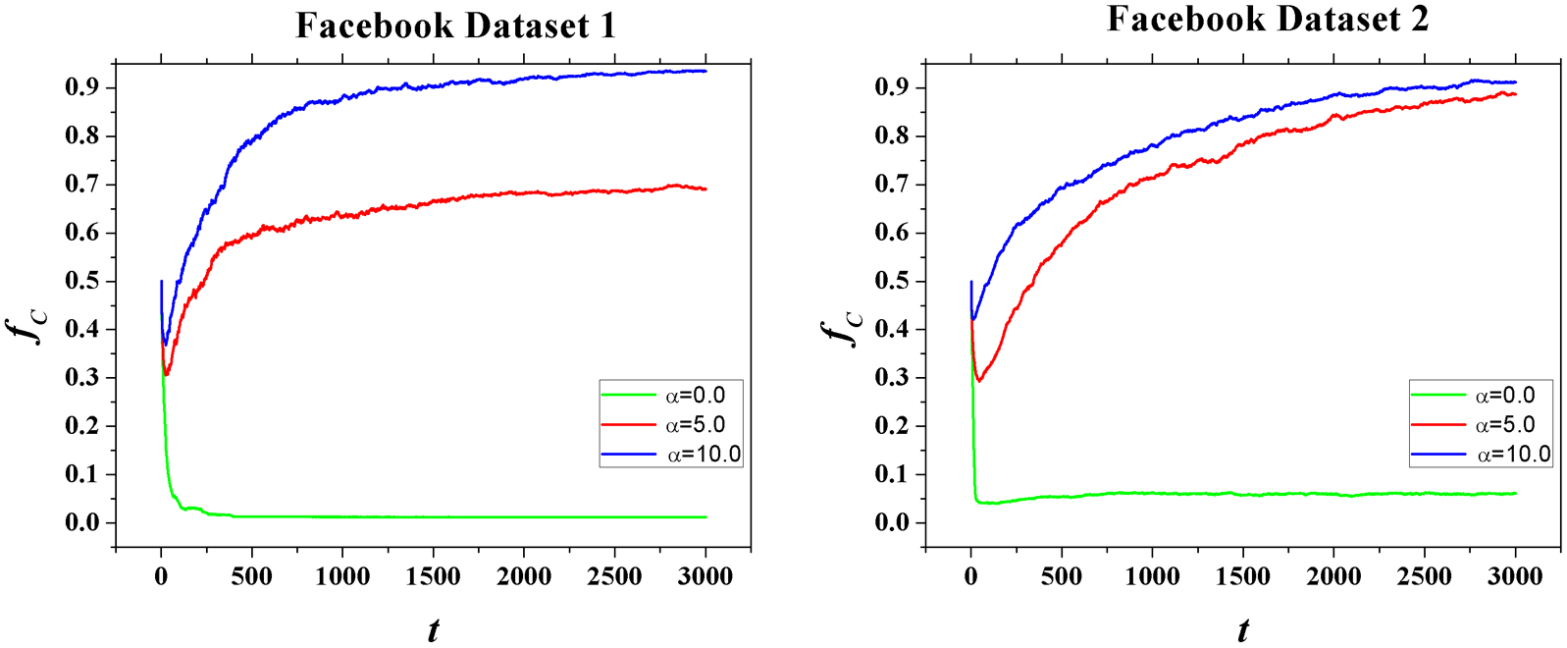
The effect of investment preferences 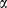 on the evolution of cooperation. 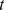
 denotes time steps. We set 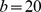 and 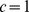
here. The results show that cooperation is prohibited if the investment preference is weak (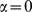). Extremely strong 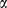 leads to global cooperation.

Fig. 4. shows the different evolutionary processes of cooperation under different 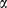. It is obvious that strong preferences on best friends have dramatically promoted cooperation. Since greater 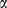 strengthens the interactions between good friends, it is natural to infer that such strong relations play key roles on the maintenance of cooperation in social networks. To explain such results, we consider an extreme scenario where the preferences on strong relations are very strong (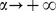). Under this setting, players will only invest in their best friend and their nodding friends get almost nothing from them, i.e. the interactions between best friends determine the processes of evolution. Moreover, it is worth noting here that most best friends are mutual in our empirical datasets. We observe 130 pairs of mutual best friends in dataset 1 and 1118 pairs in dataset 2. This is in agreement with the classical social network theory arguing that reciprocity is a key feature of social relations in real populations [33], [34]. Individuals tend to behave towards someone in the manner in which they behave toward him. If the best friend of A is B, then A is very likely to be the best friend of B. Therefore, the interactions between mutual best friends determine the direction of evolution in real social networks. Consider a pair of mutual best friends 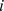 and 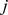, there are four possibilities for their strategic portfolios: (1) 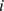 and 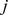 are both cooperators; (2) 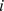 is a cooperator and 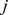 is a defector; (3) 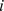 is a defector and 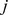 is a cooperator; (4) 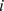 and 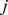 are both defectors. In the last three situations, it is obvious that mutual defection is the only equilibrium for 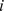 and 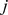. Because even in (2) and (3), the defector will get much more support from his best friend and thus his payoff will become significantly higher than that in classical models where 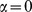. Defection is favored if at least one of the mutual best friends adopts D. However, if the two mutual best friends adopt mutual cooperation, the overall payoff of this cooperation ally will become extremely large. The two friends will support each other with all of their investments and each of the friends receives significantly greater benefits. If 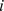 and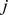 are mutual best friends and 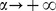, the total payoff of player 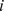 and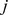 of the four scenarios can be calculated as follows:

### Scenaro 1

i and j are mutual cooperators:
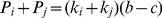
(5)


Let 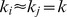:
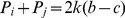
(6)


### Scenario 2 and 3

i (j) is a cooperator and j (i) is a defector:
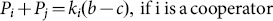
(7)

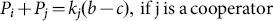
(8)


Since 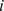 and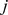 are mutual best friends, we can infer that most of the friends of player 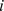 and 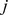 overlap (see equation 1). Therefore, 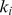 and 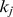 are highly correlated with each other. Let 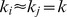, we have:
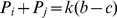
(9)


### Scenario 4

i and j are mutual defectors:
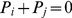
(10)


In scenario 1, mutual defection produces zero benefit since neither of the friends is willing to invest in their counterparts.

**Figure 5:**
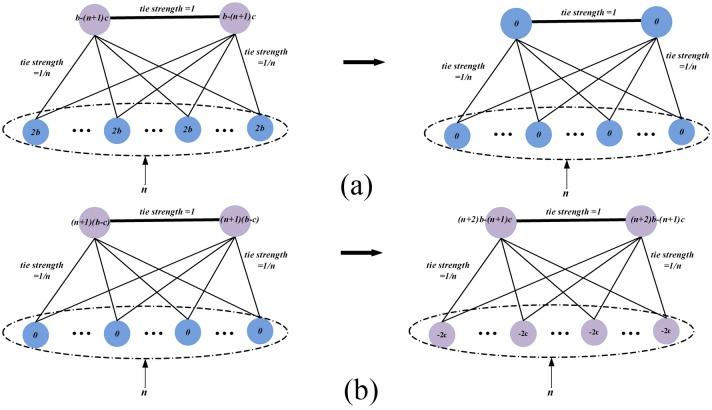
A simple illustration of the influence of 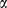 on the evolution of cooperation in PDG. Here, pink nodes denote cooperators and blue nodes denote defectors. All the two top nodes’ friends overlap, therefore the tie strength between them equals one. All other relations have a tie strength equals 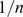. We set the two mutual best friends as cooperators initially. (a) When 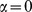, the game is a classical PDG. The two cooperators get a payoff of 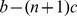 and all the defectors get 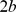. Therefore, cooperators will imitate the strategy of defectors and defection becomes prevalent; (b) When 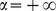, the two cooperators will invest all their investments to each other. Both cooperators get 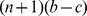 and all defectors get 0. Therefore, in the next round, all defectors will adopt C and cooperation becomes prevalent.

**Figure 6:**
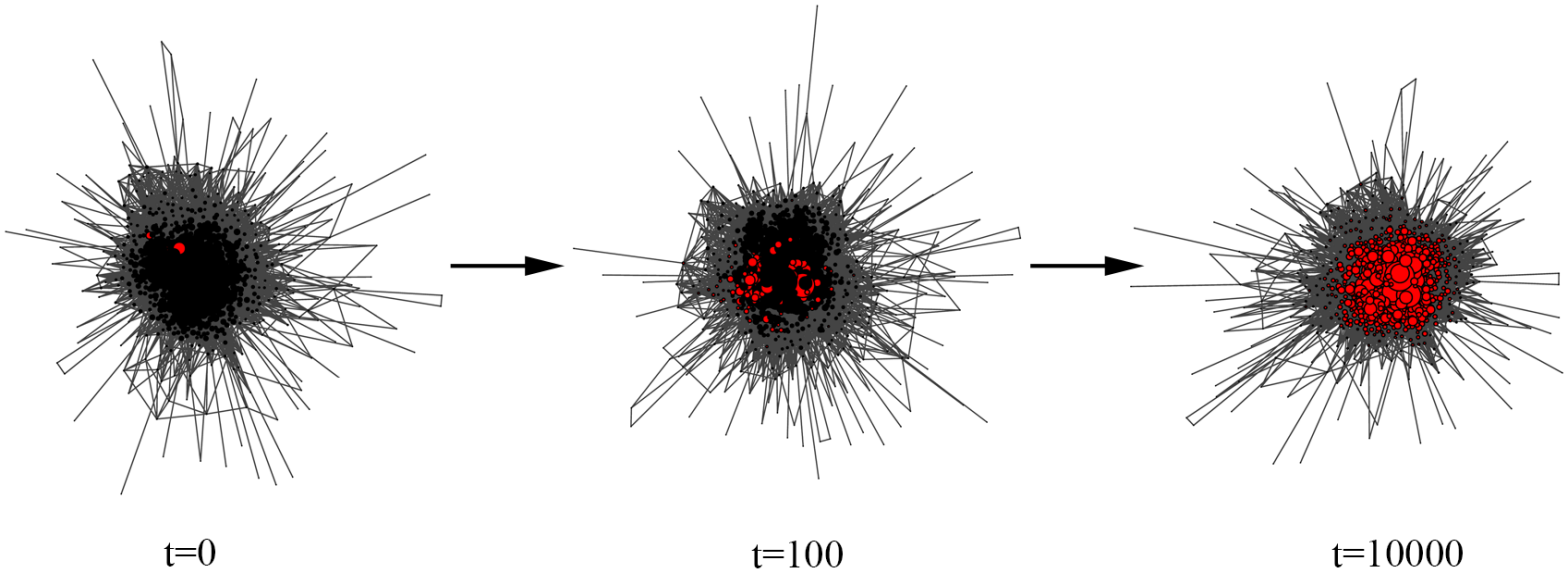
The evolution of cooperation in Facebook dataset 1 with denoting only two pairs of best friends (four cooperators) as cooperators initially. The size of each node is related to its degree. Red nodes represent cooperators and black nodes represent defectors. We set 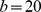, 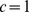 and 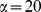. The game is repeated for 10000 times. The fraction of cooperators becomes 1 after 2000 time steps.

The above equations show that if a cooperator has a relatively large degree and his best friend is also a cooperator (scenario 4), they will produce the greatest payoff and form a extremely strong cooperation ally. This ally is hard to be invaded by other defectors. Instead, it tends to transform its neighboring defectors into cooperators. Figure 5 illustrates a simplified situation where two best friends are mutual cooperators. The figure shows when 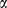 is large, the two best friends support each other with all their investments. Their payoff becomes so large that they will turn all their neighbors into cooperators. To validate this analysis, we randomly select two pairs of mutual best friends from dataset 1 as cooperators and leave all other players as defectors initially before the game starts. The simulation result (see Fig. 6.) shows that only two pairs of mutual cooperators can lead to global cooperation in a real social network.

As suggested by the experiment conducted by Harrison et. al., mutual good friends are more willingly to cooperate with each other and invest more to each other [19]. This finding indicates that instead of randomly distributing equal percentage of cooperators and defectors before the game starts, it is human nature that players will preferentially cooperate with their good friends. Such born preference facilitates the formation of cooperative clusters from the very beginning, and thus promotes cooperation. Moreover, the existence of such preferences implies that a player can play different roles in the same round. In our proposed model, the parameter 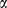 is a tunable parameter controlling this preference. When 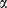 is extremely large, a player will be a cooperator to his best friend (invest all) and a defector to all other friends (investment nothing) in the same round. When 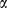 is zero, a player can only choose to be either a cooperator or a defector in a given round and this identity is the same to all his friends. The presence of diverse social relations endows players with different roles when playing against friends of different tie strengths and this diversity promotes cooperation in PDG.

**Figure 7:**
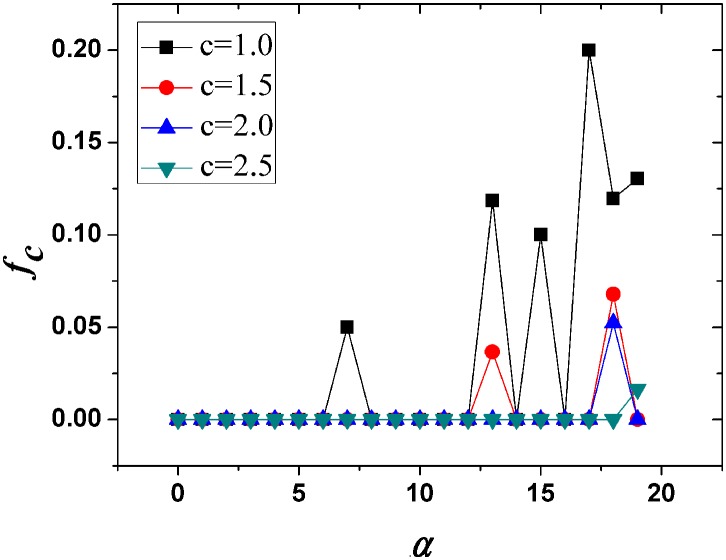
An identical simulation test is performed on a scale free BA network. The network has exactly the same number of nodes and edges as that in Facebook dataset 1. We observe no significant influence of 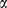 on the evolution of cooperation.

Further, we argue that the introduction of real social network datasets has very important implications on the understanding of cooperative behaviors in reality. Our study shows the limitations of traditional complex network models in analyzing the effect of diverse social relations in PDG. Despite that artificial network models indeed capture some important features of networks in reality, such as heterogeneous degree distribution, small world property, etc., they are incapable of reflecting some crucial features of social relations. To sum up, the deficiencies of complex network models in depicting social relations lie in following aspects: First, complex network models fail to represent the diversity of social relations. As we illustrated in figure 1, the distribution of tie strengths in real networks should be heterogeneous if we use equation 1 to calculate tie strengths. However, none of the current network generating models is able to produce such networks; Second, most social relations are reciprocal in reality. If A’s best friend is B, then B’s best friend is also very likely to be A. We didn’t find this feature in artificial networks. Third, we cannot generate an artificial network and randomly assign tie strengths to its ties. Numerous previous studies have already confirmed the positive relationship between the strength of the tie and the extent of overlap of social relations between the two individuals. Randomly assigning tie strength values cannot reflect this structural feature. The above three problems make artificial network models incapable of presenting the features of social ties in reality. Since player interactions are carried out through social ties, the diverse nature of social relations will inevitably pose significant influence on players’ behavioral patterns in games. Neglecting such important features will hinder our perception of the evolutionary games. The simulation results on BA networks also confirm our argument (see figure 7).

## Conclusion

The existence of social relations is the fundamental premise of the interactions between individuals in social networks. It is suggested that the intrinsic nature of the social relationships has a significant impact on the behavioral patterns of the player level interactions [35]–[38]. Although the classical hypothesis of homo economicus argues that the only pursuit of a selfish player is to maximize his own benefits [39], [40], empirical experiments show that players do exhibit some degree of altruism in reality and the extent of this altruism is positively related to the tie strength between the two players [19]. A player is more cooperative when playing with a good friend and more selfish when playing with a nodding friend. In other words, the diverse distribution of tie strengths endows players with different roles when facing different opponents and such relational diversity will inevitably influence the evolution of cooperation in PDG. Previous studies discovered that social diversity promotes cooperation in evolutionary games [41]. The effects of the diversity of degree [13], [14], wealth [17], multiplication factors [42], [43] etc. are proven to be facilitative to the emergence of cooperation in PDG. However, no investigation on the effect of diverse social relations has been conducted so far. Our research tries to fill this gap by proposing a relation based investment mechanism in PDG. Based on the empirical findings in human experiments, we propose a model arguing that a player is not likely to distribute his investments equally to his friends in reality. His good friends tend to get more from his investments and those nodding friends usually get little. We prove that this relation based preference promotes cooperation since cooperation allies between good friends produce large payoffs to resist the invasion of defectors; In fact, this investment strategy infers the fact that players can play different roles while facing friends with different tie strengths. They are more likely to cooperate (defect) with their good (ordinary) friends; Moreover, this research also highlights the importance of introducing real social network data into the analysis of the networked PDG. We show that neither of the classical complex network models is suitable to explain the effect of tie strength on the evolution of cooperation in real social networks, because real social networks exhibit diverse tie strength distributions and reciprocal social relations which cannot be produced by artificial network models. The conclusions of this research have very important theoretical and practical implications and we believe that the introduction of diverse social relationships will significantly improve our understandings on the evolution of cooperation in PDG.
